# Surgical aspects of valve replacement in carcinoid heart disease

**DOI:** 10.1111/jocs.15169

**Published:** 2020-10-30

**Authors:** Anders Albåge, Marco Montibello

**Affiliations:** ^1^ Department of Cardiothoracic Surgery and Anesthesiology University Hospital Uppsala Sweden; ^2^ Department of Surgical Sciences Uppsala University Uppsala Sweden

**Keywords:** carcinoid heart disease, cardiovascular pathology, valve replacement

## Abstract

Tricuspid and pulmonary valve replacement in patients with advanced carcinoid heart disease (CaHD) reduces right heart failure and improves prognosis. The surgical literature is limited concerning description of technical aspects of valve replacement in CaHD. Although a dedicated multidisciplinary care is required for these frail patients, optimization of surgical technique is important and may lead to better postoperative outcomes.

## 1 INTRODUCTION

Carcinoid heart disease (CaHD) is a complication of metastasizing neuroendocrine tumors (NETs), which are rare slow‐growing malignancies usually originating in the small intestine. High levels of circulating serotonin and other vasoactive substances released by the tumors cause, by complex processes, formation of plaques and fibrosis of the tricuspid (TV), and pulmonary valves (PV). Advanced CaHD of the right‐sided valves leads to severe regurgitation and/or stenosis, eventually causing progressive right heart failure and worsening of the patient's oncological prognosis.^1^ Valve surgery in CaHD patients has been shown to reduce right heart failure, increase functional capacity, allow for more aggressive oncological treatment and improve long‐term prognosis. Several smaller series of 19–32 operated patients have been reported,^2^, ^3^, ^4^ but the largest one is the 30‐year experience from Mayo Clinic with 240 CaHD patients.^5^, ^6^ In this study, 30‐day mortality after surgery was 5%, while 2‐year survival was 60%, with most patients succumbing to progressive tumor burden. Despite the prevalent outcome reporting, there is a remarkable shortage in the surgical literature concerning the technical aspects and optimization of surgical details of valve replacement in these rare patients. Tricuspid valve replacement (TVR) is always a part of these operations, and most often the pulmonary valve has to be replaced concomitantly (PVR). However, TVR and PVR are quite uncommon procedures in routine adult cardiac surgical practice. Patients with severe CaHD are frail and optimal surgical technique may lead to better outcomes. The focus of the current report is not to demonstrate patient results, but to describe some of the surgical technical issues concerning TVR and PVR in CaHD, based on our recent experience of 17 cases in the past 4 years (Table 1). The need for informed consent was waived by the local ethics committee.

**Table 1:** Patients undergoing valve surgery for CaHD 2016–2020

M + F		11 + 6
Mean age		64.2
Type of valve surgery	TVR only	2
	TVR + PVR	12
	TVR + PVR + AVrep	1
	TVR + PVR + AVR + MVR	2
Additional surgery	CABG	1
	PFO closure	2

Abbreviations: AVR, aortic valve replacement; AVrep, aortic valve repair; CABG, coronary artery bypass grafting; CaHD, carcinoid heart disease; MVR, mitral valve replacement; PFO, persistent foramen ovale; PVR, pulmonary valve replacement; TVR, tricuspid valve replacement.

## 2 PRE‐ AND PERIOPERATIVE CONSIDERATIONS

Indications for valve surgery in CaHD patients are outlined in guidelines^7^ and include progressive right heart failure with echocardiographical findings of moderate to severe insufficiency of the right‐sided valves. The TV is always involved in surgical candidates and is usually severely regurgitant, while the PV often shows a combination of stenosis and regurgitation. The decision for valve replacement should be based on a multidisciplinary evaluation of general operability in relation to oncological status and cardiac function. Timing of surgery with preoperative optimization of nutritional status and somatostatin analog treatment for carcinoid hormonal activity is essential. Studies indicate that earlier intervention rather than late improves outcomes.^7^ In our experience, the PV pathology is often underestimated on preoperative echocardiography, and a larger regurgitation may be unmasked by a higher forward flow after TVR, if leaving the PV untreated. Also, an uncorrected significant pulmonary regurgitation after TVR may lead to progressive right heart dilatation and poorer results.^8^ Thus, a low threshold is recommended for replacing the PV. The aortic or mitral valves may also be involved in 10%–15% of cases with CaHD. A previous study has shown that surgery of the left‐sided valves is not a factor for worse results and should be performed concomitantly with right‐sided valve surgery if indicated.^5^

A particular risk with CaHD patients is the occurrence of a carcinoid crisis during surgery. Anesthesia, surgery or drugs may trigger release of vasoactive hormones, causing potentially life‐threatening circulatory instability with severe hypotension and flushing.^9^ Routine therapy to counter this complication is infusion of short‐acting octreotide, started before surgery (12–24 h), continued perioperatively and for 3 days postoperatively. Furthermore, intraoperative protection of right ventricular (RV) function is key. The RV is dilated in most surgical candidates, and surgery should be performed before significant RV dysfunction develops. CaHD patients are at increased risk of bleeding during surgery, due to their oncological status, severe preoperative venous stasis, and reduced liver function. Increased attention to bleeding control is important, with regard to surgical technique, use of autologous blood recovery systems and optimization of postoperative coagulation using point‐of‐care techniques.

## 3 OPERATIVE TECHNIQUES

3.1

Both mechanical and bioprosthetic valves have been used for the right‐sided valve replacements in CaHD. However, in recent reports, bioprostheses seem to be preferred, as life‐expectancy is limited in these patients and warfarin therapy may not be well managed.^5^

3.2

After sternotomy, aortic cannulation is performed in standard fashion, although inserting the cannula more to the right side of the ascending aorta will improve exposure of the PV. Both venae cavae are snared after separate venous cannulation and commencing cardiopulmonary bypass (CPB). Triple lines for pump suction are utilized and CO_2_ wound irrigation is started. Basically, both right‐sided valves can be replaced on CPB using no or intermittent aortic cross‐clamping. In our view, however, cardioplegic arrest provides better visualization and detailed evaluation of the diseased valves, and more accurate placement of valve sutures. Before starting CPB, the planned incision on the anterior surface of the pulmonary artery (PA) is marked by a felt pen. The order of valve replacement is by surgeon preference, but we generally begin with the PV. We use only antegrade cold blood cardioplegia, every 20 min and in large amounts, for optimal protection of the RV. Retrograde cardioplegia may be insufficient in this respect.

### 3.3 Pulmonary valve

**Figure 1:**
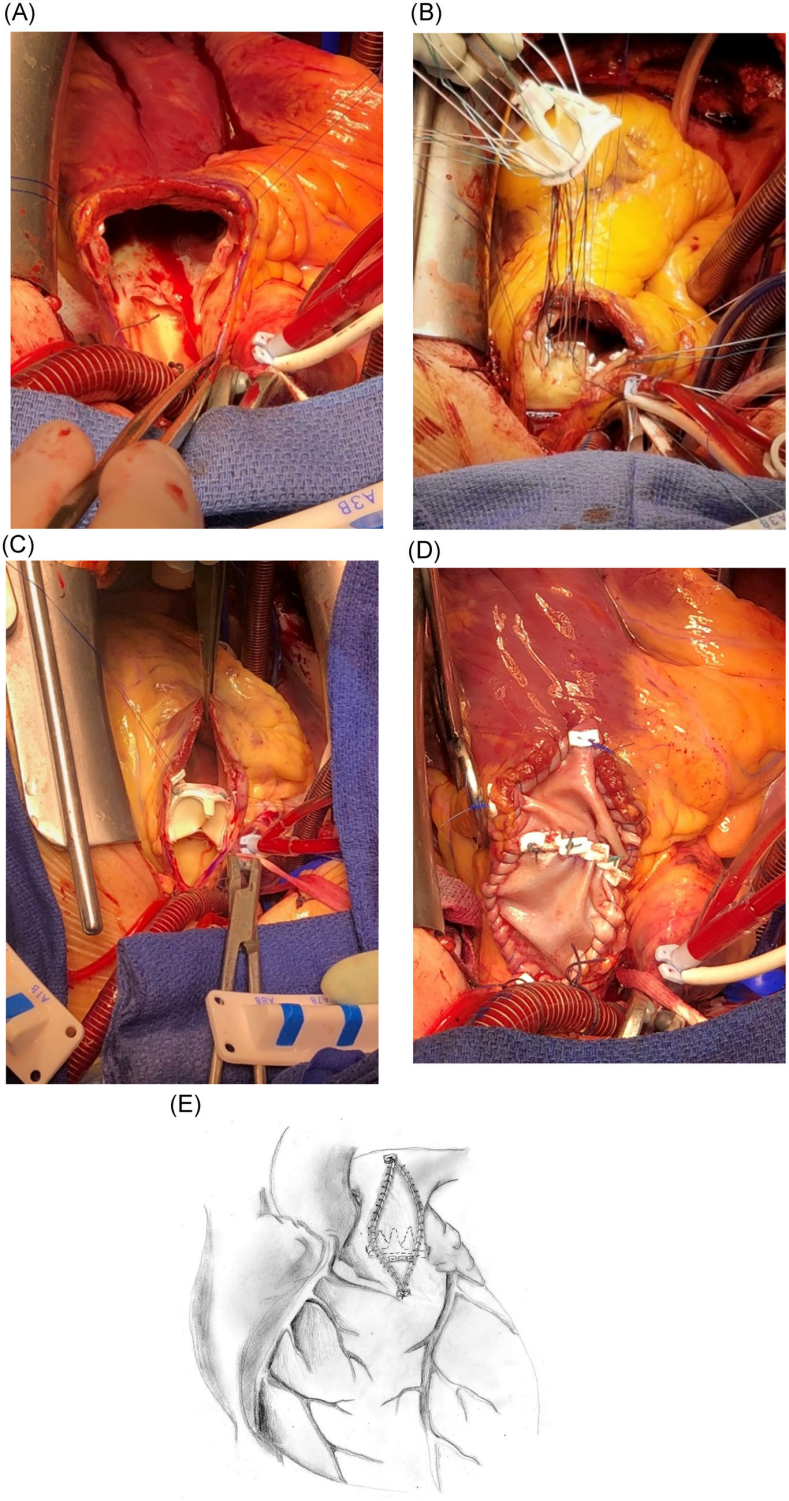
(A) Longitudinal incision from the distal PA, through the PV, into the RVOT. The exposed PV shows fibrotic cusps. (B) After excision of the PV cusps, a bioprosthesis is implanted (Carpentier–Edwards Magna Ease), conforming to the natural PV commissures. (C) The anterior portion of the bioprosthesis is left unanchored. (D and E) Enhancement of PA and RVOT dimensions is created with a bovine pericardial patch (A and D, same patient; B and C, different patients)

An incision is made in the proximal PA and extended backwards across the PV and 3–4 cm into the right ventricular outflow tract (RVOT; Figure 1A). The PV is exposed by stay‐sutures, evaluated, and the fibrotic cusps are excised. A bioprosthetic valve of appropriate size is measured. Pledgeted matress sutures are placed in the annulus, matching the commissures of the prosthesis with the native annular shape (Figure 1B). Approximately one‐fourth of the prosthesis is left unanchored with one commissure pointing anteriorly (Figure 1C), to enhance RVOT and PA dimensions. A bovine pericardial patch is tailored and sutured to the incision with a running polypropylene suture, starting from the PA corner and continued proximally on both sides. The patch should be wide enough not to impair the function of the inserted prosthesis, as it will be seated in a slightly different angle than the native PV. The remaining part of the prosthetic valve is anchored to the patch horizontally, either with a running suture or with additional pledgeted sutures from the sewing ring through the patch. Lastly, the remainder of the patch is sutured to the RVOT incision (Figure 1D,E).

### 3.4 Tricuspid valve

**Figure 2:**
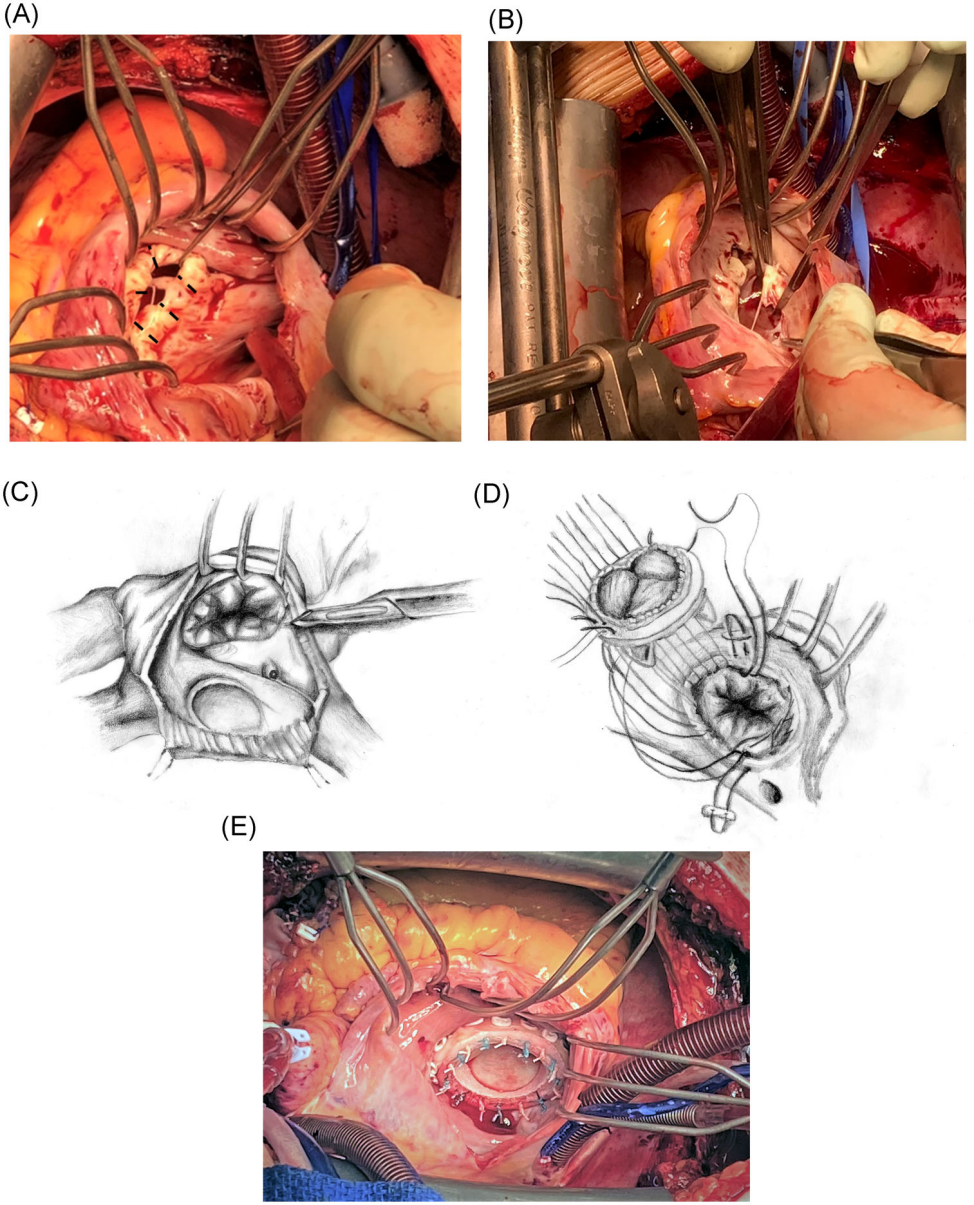
(A) Opened RA showing a fibrotic retracted TV. Suggested black lines for leaflet incisions to augment valve opening. (B and C) Multiple TV leaflet incisions are made from the free edge to the annulus respecting chordal attachments (different patient from A). (D and E) A pericardial bioprosthetic valve of largest possible size is implanted using everting sutures (Carpentier–Edwards Perimount Plus)

After opening the right atrium (RA), a methodical inspection for and closure of a persistent foramen ovale should be performed. The TV is generally fibrotic with retracted nonmobile leaflets and a narrowing of the valve opening. A prosthetic valve of the largest possible size should be implanted. Some authors advocate resection of the anterior and posterior leaflets, while leaving the septal leaflet intact with chordae. We routinely keep all leaflets, making multiple incisions from the free edge to the annular plane and leaving the chordal attachments intact to preserve tricuspid annular and RV synchrony (Figure 2A–C). This technique widens the valve sufficiently and allows for an adequately sized prosthetic valve. Occasionally, a thickened chordae clearly retracting the valve may have to be cut. We use everting atrially pledgeted sutures for anchoring the valve, passing the needles through the annulus and the body of the leaflets (Figure 2D). Caution is advised, as the annulus is frail, and deep bites in the posterior leaflet area may compromise the right coronary artery. We prefer pericardial bioprosthetic valves with a softer sewing ring, which fit better and can be tied down more gently without ripping the annular tissue. The valve should be oriented with one commissure towards the corner of the native anteroseptal commissure and another valve commissure toward the posteroseptal commissure (Figure 2E).

### 3.5 Aortic and mitral valves

In very few CaHD patients, the aortic or mitral valves are severely regurgitant in addition to the dysfunctional right‐sided valves and must be addressed with standard bioprosthetic AVR and/or MVR concomitantly. Repair of the mitral and aortic valves have also been reported. In our experience, carefully selected patients can tolerate and benefit even from quadruple valve replacement.^10^

## 4 POSTOPERATIVE COURSE

As described by others,^11^ there is a risk of complete heart block after TVR, and we routinely place permanent epicardial pacing wires on the RV and sometimes on the RA. The electrodes are placed in a subclavicular pocket on either side. In our experience, the majority of patients will not need a permanent pacemaker, but if so it may be connected in a separate procedure a few days postoperatively. CaHD patients undergoing valve surgery have a slower recovery and normally require prolonged intensive care to monitor cardiac and renal function, control of infection and carcinoid activity. Intravenous octreotide therapy is usually continued for 3 days postoperatively in collaboration with endocrine oncologists. For anticoagulation, we routinely use low‐molecular weight Heparin for 3 months, and then switch to aspirin.

## 5 CONCLUSION

TVR and PVR are the most common procedures in valvular surgery for CaHD. Although these patients are best cared for by a dedicated multidisciplinary team, optimization of surgical technique is important and may lead to improved postoperative outcomes.

## AUTHOR CONTRIBUTIONS

*Concept, data collection, and revision/approval article*: Anders Albåge and Marco Montibello. *Drafting of the article*: Anders Albåge. *Illustrations*: Marco Montibello.
